# Measuring the psychosocial consequences of screening

**DOI:** 10.1186/1477-7525-5-3

**Published:** 2007-01-08

**Authors:** John Brodersen, Stephen P McKenna, Lynda C Doward, Hanne Thorsen

**Affiliations:** 1 Department and Research Unit of General Practice, University of Copenhagen, Oester Farimagsgade 5, 24Q, Postbox 2099, DK-1014 Copenhagen, Denmark; 2 Galen Research, Enterprise House, Manchester Science Park, Lloyd Street North, Manchester M15 6SE, UK

## Abstract

The last three decades have seen a dramatic rise in the implementation of screening programmes for cancer in industrialised countries. However, in contrast to screening for infectious diseases, most cancer screening programmes only have the potential to reduce mortality; they cannot lower the incidence of cancer in a population. In fact, most cancer screening programmes have been shown to increase the incidence of the disease as a consequence of over-diagnosis. A further dilemma of cancer screening programmes is that they do not distinguish between healthy people and those with disease. Rather, they identify a continuum of disease severity. Consequently, many healthy people who have abnormal screening tests are wrongly diagnosed. Indeed, studies have demonstrated that for each screening-prevented death from cancer, at least 200 false-positive results are given. Therefore, screening has the potential to be harmful as well as beneficial. The psychosocial consequences of false-positive screening results cannot be determined by diagnostic tests or by other technical means. Instead, patient reported outcome measures must be employed. To measure the outcomes of screening accurately and comprehensively patient reported outcome measures have to capture; the nature and extent of the psychosocial consequences and how these change over time. The outcome measures used must have high content validity and their psychometric properties should be determined prior to their use in the specific population. In particular it is important to establish unidimensionality, additivity and item ordering through the application of Item Response Theory.

## Background

The history of medical screening began with that for syphilis and later for tuberculosis. Screening for infectious diseases served a dual purpose; to cure the patient and to reduce the incidence of the disease in the general population [1]. The World Health Organization (WHO) screening criteria published in 1968 highlighted major concerns with the occurrence of false-negative screening results; as people with undetected disease continue to be a source of infection [2]. The past three decades have seen a dramatic increase in the implementation of screening programmes for cancer in industrialised countries. However, in contrast to screening for infectious diseases, most cancer screening programmes only have the potential to reduce mortality; they cannot lower the incidence of cancer in a population. In fact, most cancer screening programmes have been shown to increase the incidence of the disease as a consequence of over-diagnosis or increase the incidence of conditions that are characterised as pre-cancers, dysplasia or atypical cells. [3-7]. It is important to acknowledge that by far the majority of these are harmless conditions that will never become invasive cancers [8-12].

## Discussion

Cancer screening in a general population does not distinguish between healthy people and those with disease. Rather, it identifies a continuum of disease severity [13]. For example, a recent study by Taupin et al found that 45% of participants screened for colorectal cancer had either hyperplastic (benign) or adematous (pre-cancerous) polyps which were removed [14]. Most lay people would probably see the presence of a growth as being indicative of cancer and its removal as a positive event. However, as polyps rarely become malignant, their removal could actually be viewed as over-treatment of a harmless condition.

Another dilemma with any mass cancer screening programme is that screening tests tend to have low predictive power. Consequently, many healthy people having abnormal screening tests are wrongly diagnosed (termed false alarms or "false-positive" results). Indeed, studies have demonstrated that for each screening-prevented death from cancer, at least 200 false-positive results are given [7,15,16]. In the case of mammography screening, studies have shown that the receipt of a false-positive result has substantial negative psychosocial consequences for women. These can persist for up to three years after the screening procedure [17,18]. Clearly, medical screening has the potential to be as harmful as it is beneficial [19].

In response to this dilemma the WHO-criteria for screening have recently been updated. New criteria have been added concerning ethical aspects of the screening process, the psychosocial consequences of false-positive screening results and the need for fully informed consent [20].

The psychosocial consequences of false-positive screening results cannot be determined by diagnostic tests or by other technical means. Instead, patient reported outcome (PRO) measures must be employed. To measure the outcomes of screening accurately and comprehensively, PRO measures have to capture:

• the nature of the psychosocial consequences,

• the extent of the psychosocial consequences, and

• changes in psychosocial consequences over time.

The measures used must have high content validity [21]. This means that they must both cover relevant aspects of the construct being measured and exclude issues that are irrelevant. Qualitative research has shown that abnormal and false-positive cancer screening results have a negative impact on the following psychosocial domains; anxiety, fear, mood, behaviour, sleep, sexuality and social functioning [22-26]. Unfortunately, studies reporting on psychosocial aspects of cancer screening have mostly employed questionnaires that have poor content validity and/or that have not been validated for this purpose [14,27-29].

For example, Taupin and colleagues used the SF-36 to assess the impact of screening for colorectal cancer [14]. There were several flaws in the design of this study which resulted in an underestimation of the negative consequences of the screening process. First, the authors did not test the adequacy of the content of the SF-36 for this study population. It is well established that generic PROs such as the SF-36 do not necessarily work in a consistent manner across different populations [30] and the instrument's psychometric properties should have been explored to justify its use. It is not unreasonable to suppose that the SF-36 would have low content validity in the setting of colorectal cancer screening as it does not cover many of the most important issues related to screening and because it contains a high number of irrelevant items. Taupin and colleagues recorded 30 minor and two major adverse events from the 231 colonoscopies undertaken. However, it is doubtful whether any of the SF-36 items would be capable of capturing the thoughts or feelings of a healthy person who experienced an adverse event.

A major problem with the SF-36 scales is that their items were selected in order to have high scale consistency. However, internal consistency does not ensure that a scale is unidimensional; that is, that all of the items measure a single underlying construct and so can be added together to yield a total scale score. Good internal consistency merely suggests that the items are correlated [31]. Modern PROs are required to establish unidimensionality (or, in the case of multidimensional PROs, unidimensionality of subscales), additivity and item ordering through the application of Item Response Theory (IRT) [32]. The Rasch model (an IRT model) provides formal representation of perfect measurement. Where items are shown to fit a Rasch model the measure can be shown to posses criterion-related construct validity [33], to be objective [34], sufficient [35] and, therefore, also reliable [36]. IRT evidence indicates that the SF-36 scales are not unidimensional and that items in the subscales cannot validly be added together [37].

Participants in the study (who were asymptomatic) were ineligible for inclusion if they had:

• gastrointestinal symptoms requiring attendance at a primary care physician in the previous year,

• significant co-morbidity,

• a prior diagnosis of cancer,

• previous colonic surgery or therapeutic anticoagulation.

Such a group would be expected to have a significantly better health status than that of an age matched general population. In fact, the SF-36 failed to show any such differences, confirming its lack of sensitivity. Only 37.3% of those invited chose to participate in the screening study suggesting that the sample consisted of those who were most positive about screening. Such people would be expected to underestimate any negative psychosocial experiences because of the perceived benefits of the procedure.

Taupin et al only found differences post colonoscopy on the mood domains of vitality, emotional role limitations and mental health. However, even for these domains mean scores only increased between 1.9 and 4.4 points, well below the 10 to 20 points needed for a clinically meaningful improvement in health status on the SF-36 subscales [38].

Despite the evidence presented in their paper the authors concluded that: *"Average-risk persons benefit significantly from colon cancer screening with colonoscopy, by improving in Mental Health and Vitality domains of Quality of Life"*. Such a conclusion is not justified. First, the psychosocial consequences of screening are best investigated in a randomised design. Secondly, it would be necessary to employ a PRO with good psychometric and scaling properties. It is essential that the PRO used has high content validity in order to capture the psychosocial consequences of screening accurately. Evidence of the unidimensionality of the collected data should also be reported. Finally, it is pertinent to ask whether it is ethical to give participants in screening exercises the impression that they will benefit from the process itself, given the absence of evidence supporting this conclusion and the availability of proof that false-positive results are common and have an adverse effect on well-being and health status [26,27].

## Conclusion

At present it is far from clear that cancer screening in a general population is effective. Such screening has the potential to be as harmful as it is beneficial. It is equally important to investigate the harms of a screening test as its benefits. For example, potential reductions in mortality from cancer need to be contrasted with the psychosocial consequences of false-positive screening results. When measuring the adverse effects of screening it is necessary to employ PRO's that are relevant to the population and that have good psychometric properties. It is recommended that the Rasch model should be adopted as the 'gold standard' for determining the adequacy of a PRO. Only where the data collected fit the Rasch model can they be verified as being objective, sufficient and reliable.

## Competing interests

JB and HT have conducted considerable research into the adverse impact on participants of false-positive tests in cancer screening and have developed outcome measures to determine these impacts. JB recently published a PhD thesis on this topic and is an advisor to the Danish National Board of Health on informed consent in breast cancer screening.

SPM and LCD have no competing interests.

## Authors' contributions

JB drafted the manuscript and coordinated the production of the article. SPM, LCD and HT assisted in drafting the manuscript. All authors read and approved the final manuscript.
